# Is increased myocardial triglyceride content associated with early changes in left ventricular function? A ^1^H-MRS and MRI strain study

**DOI:** 10.3389/fendo.2023.1181452

**Published:** 2023-06-22

**Authors:** Astrid Soghomonian, Anne Dutour, Nadjia Kachenoura, Franck Thuny, Adele Lasbleiz, Patricia Ancel, Robin Cristofari, Elisabeth Jouve, Umberto Simeoni, Frank Kober, Monique Bernard, Bénédicte Gaborit

**Affiliations:** ^1^ Aix-Marseille Université, INSERM, INRAE, C2VN, Marseille, France; ^2^ Department of Endocrinology, Metabolic Diseases and Nutrition, Pôle ENDO, APHM, Marseille, France; ^3^ Sorbonne Université, INSERM, CNRS, Laboratoire d’Imagerie Biomédicale, Paris, France; ^4^ Intensive Care Unit, Department of Cardiology, Assistance Publique-Hôpitaux de Marseille, Hôpital Nord, Aix-Marseille University, Marseille, France; ^5^ Department of Biology, University of Turku, Turku, Finland; ^6^ UPCET, Clinical Pharmacology, Assistance-Publique Hôpitaux de Marseille, Marseille, France; ^7^ Division of Pediatrics & DOHaD Laboratory, CHUV University Hospital and University of Lausanne, Lausanne, Switzerland; ^8^ Aix-Marseille Université, CNRS, CRMBM, Marseille, France

**Keywords:** type 2 diabetes, obesity, myocardial triglyceride content, cardiac magnetic resonance imaging, left ventricular function, feature-tracking, myocardial strain

## Abstract

**Background:**

Type 2 diabetes (T2D) and obesity induce left ventricular (LV) dysfunction. The underlying pathophysiological mechanisms remain unclear, but myocardial triglyceride content (MTGC) could be involved.

**Objectives:**

This study aimed to determine which clinical and biological factors are associated with increased MTGC and to establish whether MTGC is associated with early changes in LV function.

**Methods:**

A retrospective study was conducted using five previous prospective cohorts, leading to 338 subjects studied, including 208 well-phenotyped healthy volunteers and 130 subjects living with T2D and/or obesity. All the subjects underwent proton magnetic resonance spectroscopy and feature tracking cardiac magnetic resonance imaging to measure myocardial strain.

**Results:**

MTGC content increased with age, body mass index (BMI), waist circumference, T2D, obesity, hypertension, and dyslipidemia, but the only independent correlate found in multivariate analysis was BMI (p=0.01; R²=0.20). MTGC was correlated to LV diastolic dysfunction, notably with the global peak early diastolic circumferential strain rate (r=-0.17, p=0.003), the global peak late diastolic circumferential strain rate (r=0.40, p<0.0001) and global peak late diastolic longitudinal strain rate (r=0.24, p<0.0001). MTGC was also correlated to systolic dysfunction *via* end-systolic volume index (r=-0.34, p<0.0001) and stroke volume index (r=-0.31, p<0.0001), but not with longitudinal strain (r=0.009, p=0.88). Interestingly, the associations between MTGC and strain measures did not persist in multivariate analysis. Furthermore, MTGC was independently associated with LV end-systolic volume index (p=0.01, R²=0.29), LV end-diastolic volume index (p=0.04, R²=0.46), and LV mass (p=0.002, R²=0.58).

**Conclusions:**

Predicting MTGC remains a challenge in routine clinical practice, as only BMI independently correlates with increased MTGC. MTGC may play a role in LV dysfunction but does not appear to be involved in the development of subclinical strain abnormalities.

## Introduction

The dramatic rise in prevalence rates of obesity and type 2 diabetes (T2D) has led to an increasing risk of cardiovascular disease and death (1, 2). Epidemiological studies have shown that both obesity and T2D could directly contribute to the onset of heart failure independently of other cardiovascular risk factors (3–5), but the underlying mechanisms remain partly unknown. It is increasingly suggested that excessive deposition of triacylglycerol in cardiomyocytes may lead to lipotoxic injury due to overload of lipotoxic intermediates such as ceramides or diacylglycerol, resulting in endoplasmic reticulum stress, cardiac insulin resistance, oxidative stress, and mitochondrial dysfunction (6–8). Studies in diabetic and obese mice have demonstrated that myocardial steatosis can act locally and induce cell dysfunction and death through apoptosis, which ultimately leads to impaired left ventricular (LV) function (9, 10).

Using developments in proton magnetic resonance spectroscopy (^1^H-MRS) techniques enabling accurate non-invasive exploration of myocardial steatosis in humans (11), we and others have shown that patients with metabolic syndrome, obesity and T2D have increased myocardial steatosis (12–15). However, as access to ^1^H-MRS is limited in routine practice, studies on MTGC in humans, remain scarce and have only been carried out on small samples. Some research has found a clear association between myocardial triglyceride content (MTGC) and LV diastolic dysfunction in patients with T2D (14, 16), as in animals, but there are also conflicting results (15), and the impact of myocardial steatosis on LV function is still under debate. If the association effectively exists, then it would be relevant to determine whether MTGC is associated with early changes in LV function in order to (i) better understand the risk of heart failure in obesity and T2D and ultimately (ii) be able to identify patients who are at risk for developing cardiovascular events secondary to extreme cardiac adiposity.

LV function has long been assessed by measuring volume changes but is now widely assessed by myocardial strain which gives a more accurate measure of myocardial deformation (17). It is now recognized that myocardial dysfunction is detected earlier when using global strain rather than standard myocardial function parameters such as ventricular ejection fraction (18, 19). The development of feature tracking (FT) software has made it possible to measure systolic and diastolic strains and strain rates based on routinely-acquired cine magnetic resonance imaging (MRI) sequences (20, 21), and this technique matches well with tissue tagging, the gold standard for myocardial strain evaluation in MRI (22).

The aim of the present study was to determine the clinical and biological factors associated with increased MTGC and to establish whether there were associations between MTGC and early changes in LV function assessed by myocardial strain in a population of healthy subjects and in patients with T2D and obesity without heart failure.

## Material and methods

### Study design and participants

**Figure 1:**
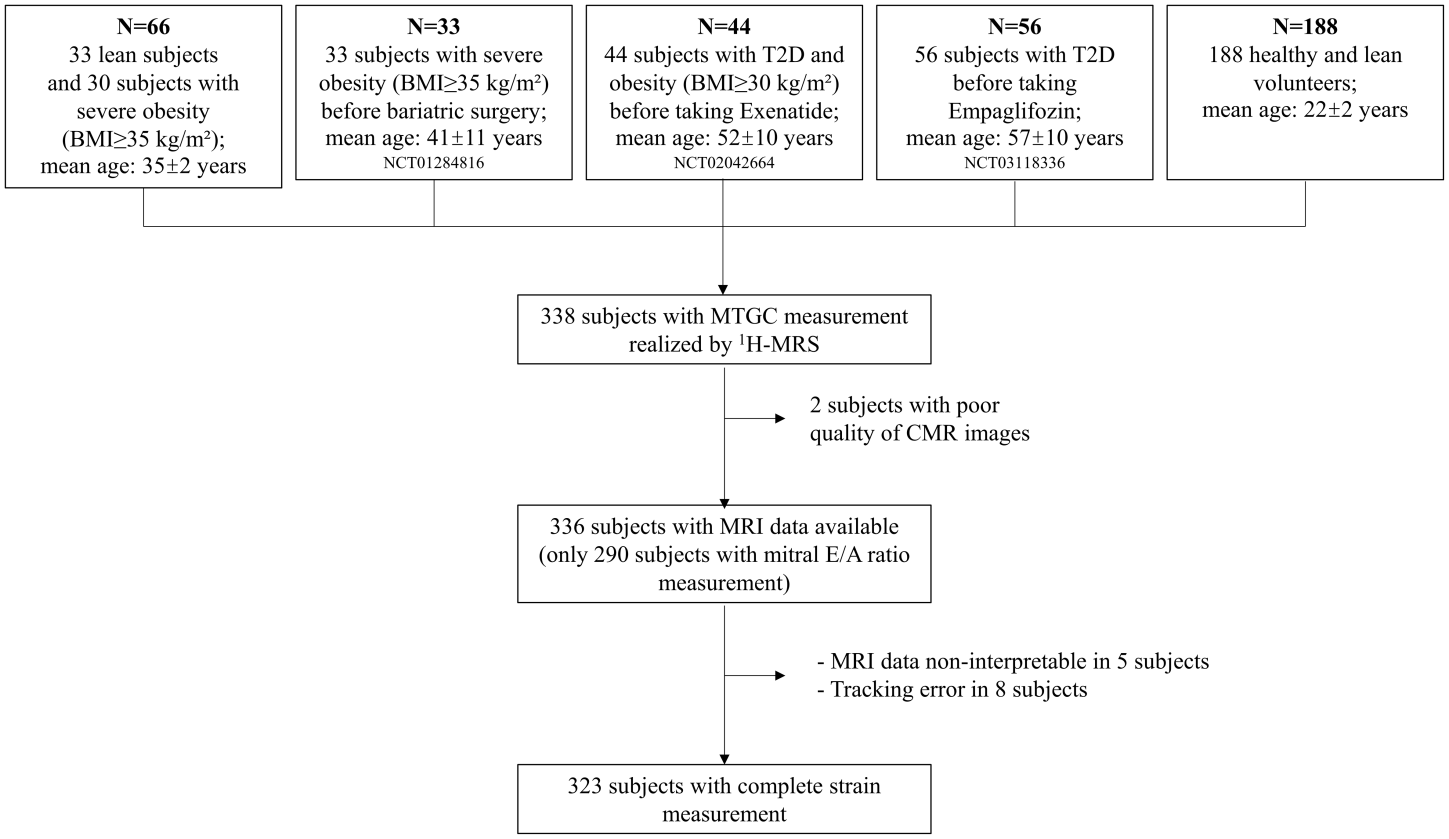
Flow chart of the cardiac resonance imaging measurements in the study population. MTGC, myocardial triglyceride content; MRI, cardiac magnetic resonance imaging; ^1^H-MRS, proton magnetic resonance spectroscopy.

Data for this retrospective, cross-sectional study was collected from five prospective cohorts managed by Assistance Publique-Hôpitaux de Marseille, the university hospital trust serving Marseille, France (12, 23–26). These cohorts included healthy lean subjects, patients with T2D, and patients with severe obesity (defined as BMI ≥35 kg/m²) (**Figure 1**). All included subjects were free of heart failure based on the European Society of Cardiology guidelines (27), including absence of symptoms and/or signs of heart failure or New York Heart Association (NYHA) class II-IV, LV ejection fraction (LVEF) ≥50%, and absence of any relevant structural heart disease and LV diastolic dysfunction. Exclusion criteria included: cardiomyopathy except coronary artery disease (CAD); primary valvular heart disease; idiopathic pulmonary artery hypertension; restrictive pericarditis; atrial fibrillation; poor image quality. Our analysis only included subjects that had a MTGC measurement performed using the same technique with proton magnetic resonance spectroscopy (^1^H-MRS), and only used data collected before the intervention (bariatric surgery or antidiabetic medication) from patients enrolled in interventional studies (NCT01284816, NCT02042664 and NCT03118336) (23–25). All protocols were approved by the institutional review board, and all subjects gave written informed consent prior to participation.

The subjects were categorized into two groups based on clinical status: a healthy group, or a non-healthy (i.e. living with T2D and/or obesity) group. They were also categorized based on MTGC quartiles in order to compare subjects with low MTGC (MTGC ≤25^th^ percentile) against subjects with high MTGC (MTGC >75^th^ percentile).

### Anthropometric and biological characterization

For all subjects, the following clinical data were collected: age, gender, history of diabetes, hypertension, smoking status, personal or familial history of coronary artery disease, dyslipidemia, and anthropometric measures, i.e. BMI, waist circumference, and hip circumference. Data from fasting blood samplings was also collected, including lipid profile (total cholesterol, triglycerides, HDL and LDL) and glucose profile (fasting plasma glucose (FPG), fasting plasma insulin (FPI) and HbA1c). Insulin resistance was assessed based on the Homeostatic Model Assessment for Insulin Resistance (HOMA-IR) calculated as follows: fasting plasma glucose (mmol/L) × fasting plasma insulin (mUi/mL)/22.5 (28).

### Cardiovascular MRI

All subjects underwent cardiovascular MRI and ^1^H-MRS to assess left ventricular structure and function and MTGC. MRI exams were performed using a 3T scanner (Siemens Verio system, Siemens Healthineers, Erlangen, Germany) using a 32-element phased array coil.

### Assessment of left ventricular morphology and function

Data on cardiac structure and function was obtained using a steady-state free precession (SSFP) cine sequence in short-axis view covering the left ventricle from base to apex. End-systolic volume (ESV), end-diastolic volume (EDV), and LV mass were measured using dedicated vendor-supplied post-processing software (Argus, Siemens Healthineers; 12,23–25). Left ventricular stroke volume (SV), cardiac output and ejection fraction (LVEF) were then calculated. SV, ESV, EDV, LV mass and cardiac output were indexed to body surface area. To determine LV diastolic function, velocity-encoded mitral valve inflow images were used to derive early (E) and late (A) peak diastolic transmitral flow velocities using an Argus workstation for quantitative flow analysis, as previously described (23).

### Proton magnetic resonance spectroscopy for MTGC quantification

To determine the molecular content of triglycerides and water in the myocardium, an ECG-gated, single-voxel, point-resolved spectroscopy sequence (PRESS) was used, as previously described (12, 23–25). The voxel was placed in the interventricular septum on cine images. Myocardial ^1^H-MRS spectra were processed offline using in-house custom software running on an IDL environment (Interactive Data Language; ITT Visual Solutions, Boulder, CO). MTGC was determined and expressed as a percentage of tissue water content (%TG = TG/water × 100) with the resonances of triglycerides and water integrated in the frequency domain and taking into account the saturation due to incomplete relaxation.

### Feature tracking for myocardial strain evaluation

LV myocardial strain was quantified on cine SSFP images using a feature tracking (FT) software developed in Matlab (MathWorks, Natick, MA) and previously employed in several studies (29–31). To initialize the FT algorithm, LV endocardial and epicardial contours were manually traced on a single time-phase of three short-axis cine slices (a basal slice immediately under the outflow tract, a mid-LV slice, and an apical slice on which the LV cavity featured throughout the cardiac cycle) and on a four-chamber cine slice. Papillary muscles were excluded from the endocardial contour. These initial contours were then automatically tracked through the cardiac cycle to enable the estimation of global systolic peaks of circumferential (GCS) and longitudinal (GLS) strains. Global systolic peak of circumferential (SRc) and longitudinal (SRl) strain rates; global early diastolic peak of circumferential (EDSRc) and longitudinal (EDSRl) strain rates; as well as global late diastolic peak of circumferential (LDSRc) and longitudinal (LDSRl) strain rates were also derived. Circumferential strains and strain rates were calculated as the average of global strains and strain rates over the three short-axis slices analyzed. In 37 subjects, automatic tracking was not accurate enough (i.e. contours failed to match to endocardial or epicardial borders throughout the cardiac cycle), so the initialization of the algorithm was repeated along with strain calculation. Even after repeating the initialization, tracking was still inaccurate in 8 subjects, who were consequently excluded.

### Statistical analysis

Statistical analysis was performed using GraphPad Prism (version 8.0.1, GraphPad Software, San Diego, CA) and R statistical software (version 4.1.3). Data were expressed as mean ± standard deviation (SD) when normally distributed, and as median (interquartile range) when non-normally distributed. A Fisher test was performed to compare qualitative variables between the two groups. Quantitative variables were compared using an unpaired t-test when the data was normally distributed, or a Mann-Whitney U test otherwise. Bivariate correlations were performed using Pearson or Spearman coefficients, as appropriate. We also performed multivariate analysis using generalized linear models to assess the effect of MTGC independently of other variables. MTGC was modeled using a beta distribution (the *betareg* package in R (32)). To take into account the multicollinearity between independent variables, we calculated the variance inflation factors (VIF) and removed the variables with the highest VIF (i.e. redundant variables) from the analysis. To study inter-operator and intra-operator reproducibility, we performed a Bland-Altman analysis and reported the mean bias and limits of agreement (mean ± 1.96·standard deviations of the differences), and computed the coefficient of variation (CV) as the standard deviation of the differences between two measures divided by their mean. Statistical significance was set at p ≤0.05.

### Reproducibility analysis

Inter-rater reproducibility was evaluated for peak strain measurements by two independent readers on four subjects. Intra-rater reproducibility was evaluated by repeating strain measurements four times in four different subjects by the same observer at intervals of a few days between each measurement. Intra-observer reproducibility was excellent, with a coefficient of variation of 2.1% for GCS and 5.5% for GLS. Inter-observer reproducibility was satisfactory, with a coefficient of variation of 1.7% for GCS and 2.3% for GLS.

## Results

### Clinical characteristics of the study population

The baseline characteristics of subjects included in this study are reported in **Table 1**. Our analysis included a total of 338 subjects, of which 29.1% had T2D, 31.9% were obese (BMI ≥30 kg/m²), 24.3% had both T2D and obesity, 20.5% had dyslipidemia, and 20% had arterial hypertension. Note that seven subjects living with T2D had coronary artery disease.

**Table 1:** Clinical and biological characteristics of the study population.

	All subjects (n=338)	Healthy subjects (n=208)	Patients living with T2D and/or obesity (n=130)	p value
Sex ratio: women: (n, %)	200 (56.9)	112 (50.9)	88 (67.2)	*0.003*
Age (years)	24 [21;49]	21 [20;24]	52 [42;60]	*<0.0001*
BMI (kg/m²)	23.9 [21;33.2]	21.6 [19.7;23.5]	37.1 [32.4;43.3]	*<0.0001*
WC (cm): - women - men	78.8 [68.5;114] 83.5 [76;103]	69 [64.6;73.9] 79 [75;85.5]	115.5 [105.3;127] 115 [107;131]	*<0.0001* *<0.0001*
Type 2 diabetes (n, %)	102 (29.1)	0	102 (77.9)	*<0.0001*
Obesity (n, %)	112 (31.9)	0	72 (54.9)	*<0.0001*
Dyslipidemia (n, %)	72 (20)	0	72 (54.9)	*<0.0001*
Arterial hypertension (n, %)	70 (20)	0	70 (53.3)	*<0.0001*
Lipid profile				
Total cholesterol (mmol/L)	4.4 ( ± 0.9)	4.4 ( ± 0.8)	4.3 ( ± 0.3)	0.07
Triglycerides (mmol/L)	0.9 [0.4;1.3]	0.8 [0.6;1.1]	1.4 [1.0;2.1]	*<0.0001*
HDL (mmol/L)	1.3 [1;1.5]	1.4 [1.2;1.7]	1.1 [0.9;1.3]	*<0.0001*
LDL (mmol/L)	2.5 ( ± 0.8)	2.5 [2.0;1.7]	2.6 [1.8;3.1]	0.16
Glucose profile				
Fasting plasma glucose (mmol/L)	4.9 [4.6;6.3]	4.7 [4.4;4.9]	7.3 [5.5;9.4]	*<0.0001*
Fasting plasma insulinemia (mUI/L)	8.2 [5.9;12.9]	7.1 [5.2;9.2]	17.3 [12.4;24.9]	*<0.0001*
HOMA-IR	1.7 [1.2;2.9]	1.5 [1.0;1.9]	4.8 [2.9;8.1]	*<0.0001*

Data expressed as mean ± standard deviation (SD) or as median [25^th^ percentile;75^th^ percentile].

BMI, body mass index; HOMA-IR, homeostatic model assessment of insulin resistance; HDL, high-density lipoprotein; LDL, low-density lipoprotein; T2D, type 2 diabetes; WC, waist circumference.

Subjects living with T2D or obesity were significantly older and had a higher BMI and higher waist circumference than healthy subjects. Among the 101 subjects living with T2D, median duration since onset of disease was 8 years [4;13] and median HbA1c was 7.38% [6.4;8.4]. In total, 91 subjects were taking biguanides, 52 were taking sulfonylureas and glinides, and 21 were taking DPP-IV inhibitors. No subjects were taking iSGLT2.

As expected, insulin resistance assessed by HOMA-IR was significantly higher in subjects living with T2D and/or obesity than in healthy volunteers (4.8 [2.9;8.1] vs 1.5 [1;1.9], p<0.0001).

Analysis of biochemical parameters showed that even when subjects living with T2D or obesity were treated by lipid-lowering drugs, they nevertheless had significantly higher plasma triglycerides (1.4 [1.0;2.1] mmol/l vs 0.8 [0.6;1.1] mmol/l, p<0.0001) and significantly lower plasma high-density lipoprotein (HDL) cholesterol (1.1 [0.9;1.3] mmol/l vs 1.4 [1.2;1.7] mmol/l, p<0.0001) than healthy subjects.

### Clinical and biological factors found to be associated with increased MTGC

**Figure 2:**
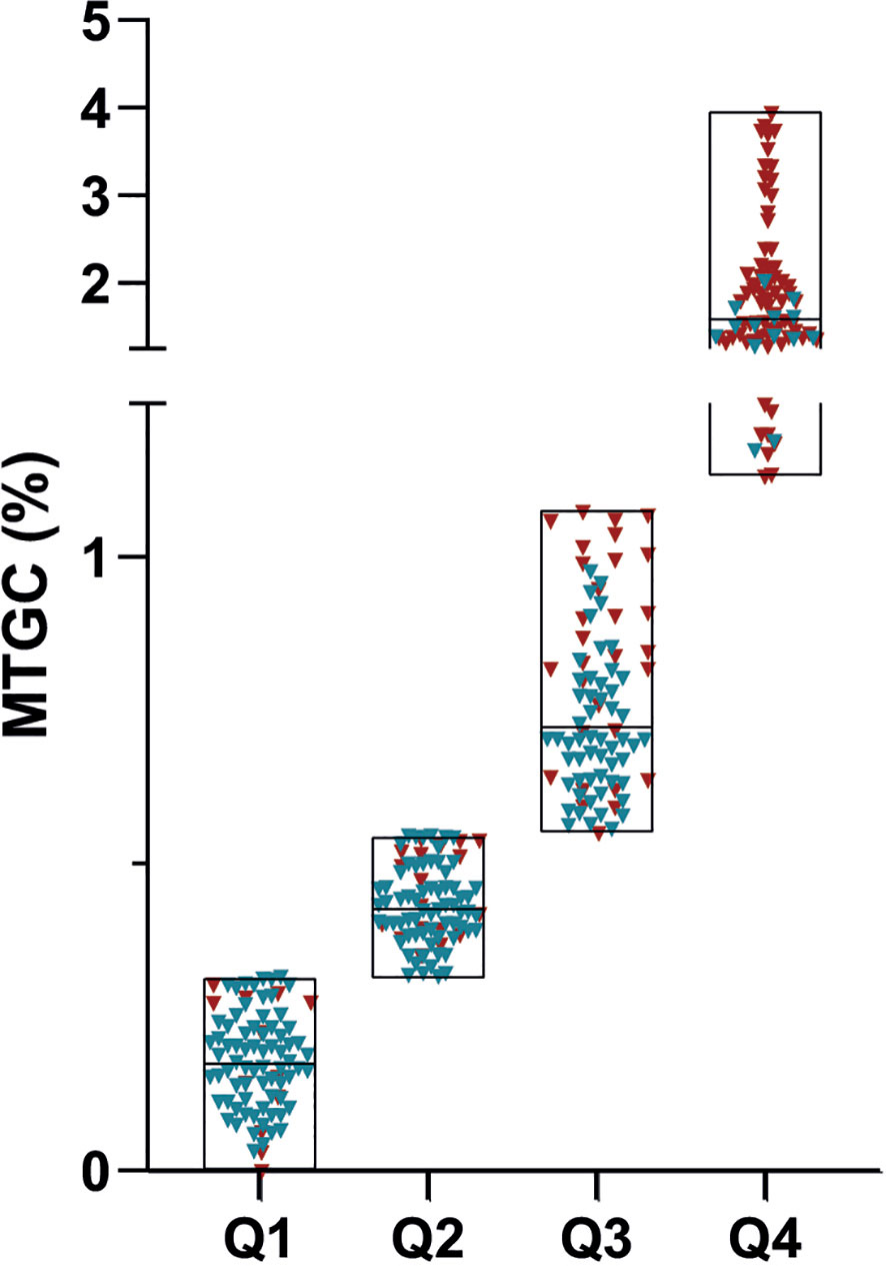
Classification of the population based on myocardial triglyceride content quartile. Q1 (n=84):0.001% to 0.314%; Q2 (n=86): 0.314% to 0.543%; Q3 (n=84): 0.543% to 1.090%; Q4(n=84): 1.090% to 3.955%;X-axis represented the median of myocardial triglyceride content divided into quartiles; Healthy subjects are represented in blue and subjects living with T2D and/or obesity are represented in red.

Participants were categorized based on MTGC quartiles (**Figure 2**), and we compared patients with low MTGC quartiles (n=84) versus patients with high MTGC quartiles (n=84) (**Table 2**). There were significantly more subjects living with T2D, obesity, hypertension and dyslipidemia (p<0.0001) in the high MTGC subgroup than in the low MTGC subgroup. Remarkably, 14 healthy subjects were in the high MTGC quartile subgroup (shown in blue on **Figure 2**) and 12 subjects living with T2D or obesity were in the low MTGC quartile subgroup (shown in red on **Figure 2**). Age, BMI and waist circumference were significantly higher in the high MTGC subgroup than in the low MTGC subgroup. Plasma triglyceride level was significantly higher and plasma HDL cholesterol was significantly lower in the high MTGC subgroup than in the low MTGC subgroup (p<0.0001).

**Table 2:** Comparison of bioclinical characteristics in subjects with low *versus* high myocardial triglyceride content.

	Low MTGC ≤ 0.31% (n=84)	High MTGC >1.09% (n=84)	p value
Clinical data			
Sex ratio: women (n, %)	46 (54.8%)	58 (69.1%)	0.08
Age (years)	22 [21;29.8]	51 [34;58.8]	*<0.0001*
BMI (kg/m²)	21.8 [19.7;24.7]	35.3 [28.9;41.5]	*<0.0001*
WC (cm): -women -men	70.5 [64.5;81.5] 78.3 [74.8;85.6]	111 [99.5;125] 110 [91.3;119.8]	*<0.0001* *<0.0001*
T2D (n, %)	8 (9.5%)	52 (61.9%)	*<0.0001*
Obesity (n, %)	11 (13.1%)	62 (73.8%)	*<0.0001*
Dyslipidemia (n, %)	5 (5.9%)	39 (46.4%)	*<0.0001*
Arterial hypertension (n, %)	5 (5.9%)	37 (44.1%)	*<0.0001*
Biological data			
Total cholesterol (mmol/L)	4.3 [3.8;4.7]	4.4 [3.5;5.1]	0.79
Triglycerides (mmol/L)	0.8 [0.6;1.2]	1.4 [1.0;2.1]	*<0.0001*
HDL (mmol/L)	1.4 [1.1;1.6]	1.1 [1.0;1.4]	*<0.0001*
LDL (mmol/L)	2.4 ( ± 0.7)	2.5 ( ± 0.8)	0.909
Fasting plasma glucose (mmol/L)	4.7 [4.7;5.1]	6.7 [5.0;8.9]	*<0.0001*
Fasting plasma insulinemia (mUI/L)	6.9 [5.0;9.9]	14.1 [8.7;21.7]	*<0.0001*
HOMA-IR	1.4 [1.0;8.6]	3.7 [1.7;6.6]	*<0.0001*

Data expressed as mean ± standard deviation (SD) or as median [25^th^ percentile;75^th^ percentile].

BMI, body mass index; HOMA-IR, homeostatic model assessment of insulin resistance; MTGC, myocardial triglyceride content; HDL, high-density lipoprotein; LDL, low-density lipoprotein; T2D, type 2 diabetes; WC, waist circumference.

As expected, subjects living with T2D had higher MTGC (1.2% [0.6;1.9]) than subjects with no T2D (0.4% [0.2;0.7]). Likewise, subjects with obesity, hypertension or dyslipidemia had higher MTGC than healthy subjects. In a subgroup analysis, comparing patients with low MTGC quartiles versus patients with high MTGC quartiles in the healthy subjects subgroup and the T2D and/or obesity patients subgroup, a higher proportion of T2D patients was found in the high MTGC quartile as expected (**Supplementary Table 4**). In univariate analysis (correlations summarized in **Table 3A**), MTGC was strongly correlated to age, BMI and waist circumference (r=0.43, p<0.0001; r=0.46, p<0.0001; r=0.45, p<0.0001, respectively) and significantly correlated to plasma triglycerides (r=0.39, p<0.0001) and plasma HDL cholesterol (r=-0.27, p<0.0001), but not with plasma low-density lipoprotein, total cholesterol, fasting plasma glucose or HOMA-IR score (**Table 3A**). In multivariate analysis, the only independent correlate of MTGC was BMI (p=0.01; R² = 0.20). Importantly, the full set of clinical and biological parameters included in the analysis only explained 20% of the MTGC variance (**Table 3B**).

**Table 3A:** Association between myocardial triglyceride content and clinical or biological parameters (univariate analysis) (n = 338).

MTGC		
	r	p value
Age (years)	0.43	*<0.0001*
BMI (kg/m²)	0.46	*<0.0001*
Waist circumference (cm)	0.45	*<0.0001*
Total cholesterol (mmol/L)	0.07	0.19
Triglycerides (mmol/L)	0.39	*<0.0001*
HDL cholesterol (mmol/L)	-0.27	*<0.0001*
LDL cholesterol (mmol/L)	0.08	0.17
Fasting plasma glycemia (mmol/L)	0.07	0.3
HOMA-IR	0.08	0.26

MTGC, myocardial triglyceride content; BMI, body mass index; HOMA-IR, homeostatic model assessment of insulin resistance; HDL, high-density lipoprotein; LDL, low-density lipoprotein.

**Table 3B:** Association between myocardial triglyceride content and clinical or biological parameters (multivariate analysis) (n=338).

MTGC dependent variable	β	p value	R²
Independent variables			
Sex (female)	0.12	0.18	0.20
Age	0.01	0.07	
BMI	0.01	*0.01*	
Waist-to-hip ratio	0.34	0.49	
T2D	0.14	0.30	
Arterial hypertension	-0.09	0.53	
HDL cholesterol	-0.48	0.10	
Triglycerides	-0.03	0.61	

BMI, body mass index; MTGC, myocardial triglyceride content; T2D, type 2 diabetes.

### Cardiac magnetic resonance imaging

As shown in the flowchart (**Figure 1**), we only had cardiac function data available for 336 subjects, due to technical issue such as poor quality of MRI data. Velocity-encoded transmitral inflow images were readable and analyzable in 290 subjects. Of the remaining 336 subjects for which we had MRI images, we were only able to perform strain measurements on 323 subjects, as the MRI data was not interpretable in 5 subjects and the FT algorithm was unable to accurately execute the tracking in 8 subjects (**Figure 1**).

### Cardiac characteristics

As patients living with T2D or obesity were significantly older and had a higher BMI than the healthy subjects, all subsequent analyses were adjusted for age, gender, and BMI. Subjects living with T2D or obesity had a significantly higher concentricity index and higher LV mass (p<0.0001) and significantly lower cardiac index, ventricular volumes (ESV, SV, EDV), mitral peak E-wave velocity and E/A ratio (p<0.0001) than healthy subjects (**Supplementary Table 1**). Systolic and diastolic strains were also significantly different between healthy and non-healthy subjects (**Supplementary Table 2**). Global peak systolic longitudinal strain (GLS) tended to be lower in T2D/obese subjects than in healthy subjects (-16.8% [-19.7;-14.8] vs -17.8% [-19.8;-15.9], p =0.07). Global peak late diastolic circumferential strain rate (LDSRc) and global peak late diastolic longitudinal strain rate (LDSRl) were significantly higher in T2D and/or obese subjects and global peak early diastolic circumferential strain rate (EDSRc) was significantly lower (p<0.0001) compared to healthy subjects (**Supplementary Table 2**).

### Impact of MTGC on cardiac characteristics

**Figure 3:**
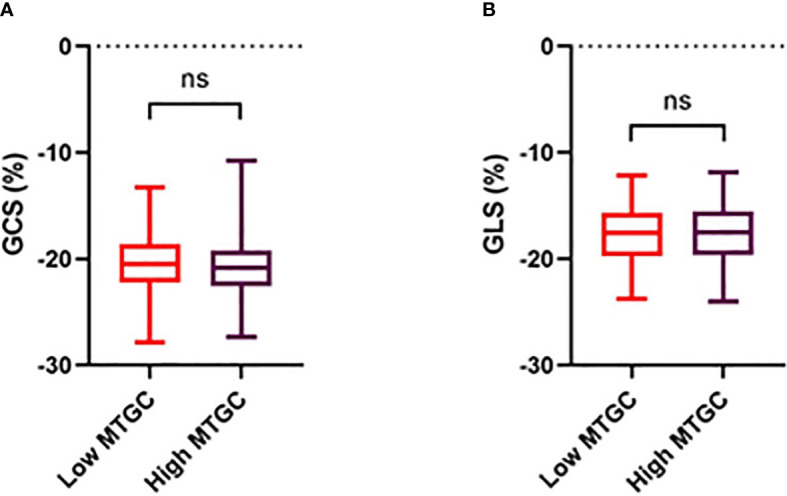
Comparison of circumferential **(A)** and longitudinal **(B)** strain between high and low MTGC subgroups. GCS, global peak systolic circumferential strain; GLS, global peak systolic longitudinal strain; ns, non significant.

**Figure 4:**
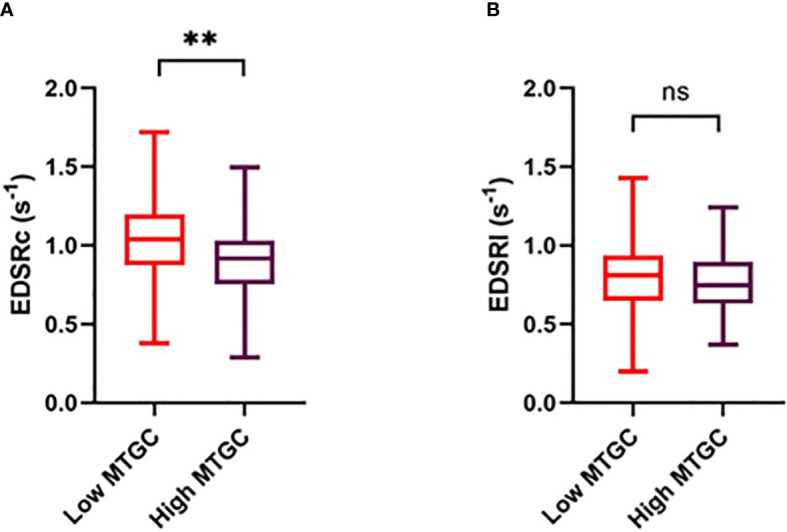
Comparison of early diastolic circumferential **(A)** and longitudinal **(B)** strain rate between high and low MTGC subgroups. EDSRc, global peak early diastolic circumferential strain rate; EDSRl, global peak early diastolic longitudinal strain rate; ns, non significant; **p<0.001.

**Figure 5:**
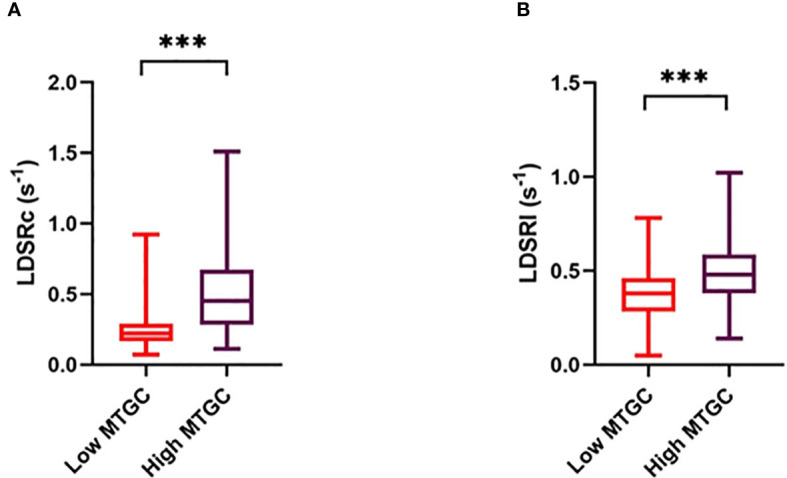
Comparison of late diastolic circumferential **(A)** and longitudinal **(B)** strain rate between high and low MTGC subgroups. LDSRc, global peak late diastolic circumferential strain rate; LDSRl, global peak late diastolic longitudinal strain rate; ***p<0.0001.

Systolic and diastolic LV volumes (ESV, SV, EDV), peak E-wave velocity and mitral E/A ratio were significantly lower in the high MTGC subgroup than in the low MTGC subgroup (p<0.0001) (**Table 4**). For systolic strain parameters, no difference was found between the two subgroups (**Figure 3**, **Supplementary Table 3**). For diastolic strain rate parameters, EDSRc was significantly lower in the high MTGC subgroup than in the low MTGC subgroup (**Figure 4**), and LDSRc and LDSRl were significantly higher in the high MTGC subgroup than in the low MTGC subgroup (**Figure 5**).

**Table 4:** Comparison of conventional left ventricular parameters between subjects with low *versus* high myocardial triglyceride content.

	Low MTGC ≤ 0.31% n=84	High MTGC >1.09% n=84	p value
Cardiac geometry			
LV mass (g)	96.1 [73.5;123.3]	106.4 [86.7;127.9]	0.05
LV mass index (g/cm²)	53.9 ( ± 11.5)	54.5 ( ± 13.1)	0.78
Conventional LV function parameters			
LV Mass/EDV (g/mL)	0.69 [0.60;0.76]	0.89 [0.75;1.04]	*<0.0001*
Cardiac output (L/min)	6.2 ( ± 1.4)	6.1 ( ± 1.5)	0.76
Cardiac index (L/min/m²)	3.4 ( ± 0.6)	3.1 ( ± 0.7)	*0.002*
Conventional LV systolic function parameters			
LVEF (%)	65.1 ( ± 6.7)	67.6 ( ± 8.3)	*0.04*
ESV (mL)	46.6 [35.6;61.3]	37.1 [30.2;50.2]	*0.0006*
ESV index (mL/m²)	26.4 [21.8;32.3]	18.7 [15.6;25.1]	*<0.0001*
SV (mL)	92 ( ± 23)	81.8 ( ± 19.8)	*0.003*
SV index (mL/m²)	50.7 ( ± 9.4)	41.3 ( ± 9.3)	*<0.0001*
Conventional LV diastolic function parameters			
EDV (mL)	136.8 [115.3;171.2]	118.2 [106.5;144.7]	*0.0008*
EDV index (mL/m²)	77.9 ( ± 14.4)	62 ( ± 14.6)	*<0.0001*
Peak E velocity (cm/s)	71 ( ± 13.5)	59 ( ± 15.4)	*<0.0001*
Peak A velocity (cm/s)	32.7 ( ± 9.3)	42.4 ( ± 14.4)	*<0.0001*
Mitral E/A ratio	2.2 [1.7;2.8]	1.3 [1.1;1.9]	*<0.0001*

Data expressed as mean ± standard deviation (SD) or as median [25^th^ percentile;75^th^ percentile].

MTGC, myocardial triglyceride content; LV, left ventricular; LVEF, left ventricular ejection fraction; SV, stroke volume; ESV, end-systolic volume; EDV, end-diastolic volume.

In a subgroup analysis, comparing patients with low MTGC quartiles versus patients with high MTGC quartiles in the healthy subjects subgroup and the T2D and/or obesity patients subgroup, only ESV and EDV were still significantly lower in high MTGC subjects in the T2D and/or obesity patients subgroup (**Supplementary Table 5**).

### Is MTGC associated with functional and structural LV parameters?

MTGC was significantly correlated with conventional LV diastolic function parameters, i.e. EDV, EDV index, peak E-wave velocity, peak A-wave velocity, and mitral E/A ratio, and with conventional LV systolic function parameters, i.e. LVEF, ESV, ESV index, SV and SV index. MTGC was also correlated with LV mass but not with LV mass index (**Table 5A**). MTGC was also positively correlated with the LV concentric remodeling index (LV mass/EDV ratio) (r=0.32, p<0.00001). To study the association between LV function and MTGC, we performed multivariate analysis with each of the cardiac parameters correlated to MTGC in univariate analysis entered as a dependent variable, and then with MTGC, gender, age, BMI, hypertension and T2D entered into the model as independent variables.

**Table 5A:** Association between myocardial triglyceride content and structural and functional LV parameters (univariate analysis).

MTGC		
	r	p value
**LV mass (g)**	0.12	*0.03*
**LV mass index (g/cm²)**	-0.01	0.83
**LV mass/EDV (g/mL)**	0.32	*<0.0001*
**Cardiac output (L/min)**	0.02	0.75
**Cardiac index (L/min/m²)**	-0.15	*0.006*
**LVEF (%)**	0.15	*0.005*
**ESV (mL)**	-0.20	*0.0003*
**ESV index (mL/cm²)**	-0.34	*<0.0001*
**SV (mL)**	-0.13	*0.02*
**SV index (mL/cm²)**	-0.31	*<0.0001*
**EDV (mL)**	-0.17	*0.002*
**EDV index (mL/cm²)**	-0.36	*<0.0001*
**Peak E velocity (cm/s)**	-0.25	*<0.0001*
**Peak A velocity (cm/s)**	0.26	*<0.0001*
**Mitral E/A ratio**	-0.35	*<0.0001*
**GCS (%)**	-0.10	0.07
**GLS (%)**	0.009	0.88
**SRc (s^-1^)**	-0.13	*0.02*
**SRl (s^-1^)**	-0.12	*0.04*
**EDSRc (s^-1^)**	-0.17	*0.003*
**LDSRc (s^-1^)**	0.40	*<0.0001*
**EDSRl (s^-1^)**	-0.08	0.15
**LDSRl (s^-1^)**	0.24	*<0.0001*

MTGC, myocardial triglyceride content; LV, left ventricular; LVEF, left ventricular ejection fraction; SV, stroke volume; ESV, end-systolic volume; EDV, end-diastolic volume; GCS, global peak systolic circumferential strain; GLS, global peak systolic longitudinal strain; SRc, global peak systolic circumferential strain rate; SRl, global peak systolic longitudinal strain rate; EDSRc, global peak early-diastolic circumferential strain rate; EDSRl, global peak early-diastolic longitudinal strain rate; LDSRc, global peak late-diastolic circumferential strain rate; LDSRl, global peak late-diastolic longitudinal strain rate.

MTGC remained independently associated to LV volumes (EDV, EDVi, ESV and ESVi) and to LV mass (**Table 5B**). Peak E-wave velocity also remained significantly associated with MTGC in this model, but with a positive coefficient (β=2.94, p=0.015, R²=0.44). Furthermore, the LV concentric remodeling index (LV mass/EDV) was no longer significantly associated with MTGC (**Table 5C**).

**Table 5B:** Association between LV mass and clinical parameters (multivariate analysis) (n=338).

LV mass dependent variable	β	p value	R²
Independent variables			
Sex (Male)	40.0	*<0.0001*	0.58
Age	-0.5	*0.0001*	
BMI	2.0	*<0.0001*	
MTGC	-6.1	*0.002*	
T2D	17.7	*0.0003*	
Arterial hypertension	9.4	*0.03*	

BMI, body mass index; MTGC, myocardial triglyceride content; T2D, type 2 diabetes.

**Table 5C:** Association between myocardial triglyceride content and structural and functional LV parameters (multivariate analysis).

MTGC			
	β	p value	R²
**LV mass (g)**	-6.05	*0.002*	0.58
**LV mass/EDV (g/mL)**	0.003	0.98	0.44
**Cardiac index (L/min/m²)**	-0.01	0.80	0.20
**LVEF (%)**	1.33	*0.03*	0.07
**ESV (mL)**	-3.94	*0.002*	0.31
**ESV index (mL/cm²)**	-1.63	*0.01*	0.29
**SV (mL)**	-2.85	0.07	0.37
**SV index (mL/cm²)**	-0.78	0.30	0.37
**EDV (mL)**	-6.40	*0.005*	0.45
**EDV index (mL/cm²)**	-2.19	*0.04*	0.46
**Peak E velocity (cm/s)**	2.94	*0.02*	0.44
**Peak A velocity (cm/s)**	0.92	0.34	0.23
**Mitral E/A ratio**	0.09	0.11	0.44
**SRc (s^-1^)**	-0.04	*0.02*	0.09
**SRl (s^-1^)**	-0.05	*0.0003*	0.13
**EDSRc (s^-1^)**	0.04	*0.02*	0.37
**LDSRc (s^-1^)**	0.02	0.17	0.55
**LDSRl (s^-1^)**	0.03	*0.04*	0.18

MTGC, myocardial triglyceride content; LV, left ventricular; LVEF, left ventricular ejection fraction; SV, stroke volume; ESV, end-systolic volume; EDV, end-diastolic volume; SRc, global peak systolic circumferential strain rate; SRl, global peak systolic longitudinal strain rate; EDSRc, global peak early-diastolic circumferential strain rate; LDSRc, global peak late-diastolic circumferential strain rate; LDSRl, global peak late-diastolic longitudinal strain rate.

Each structural and functional LV parameter was analyzed as a dependent variable with the same model as shown in **Table 5B**.

For each analysis, only the standardized regression coefficient of the independent variable MTGC is presented.

### Is MTGC associated with early changes in LV function?

Regarding systolic strain parameters, MTGC was not correlated with GLS (r=0.009, p=0.88). Regarding diastolic strain parameters, MTGC was negatively correlated with EDSRc (r=-0.17, p=0.003) and positively correlated with LDSRc and LDSRl (r=0.40, p<0.0001; r=0.24, p<0.0001, respectively) (**Table 5A**).

Multivariate analysis showed that the diastolic strain parameters, both EDSRc and LDSRl remained independently associated with MTGC (β=0.04, p=0.015, R²=0.37; β=0.03, p=0.041, R²=0.16, respectively) even after adjusting for age, gender, BMI, hypertension, and T2D (**Table 5C**).

## Discussion

To the best of our knowledge, this is the first study using MRI to assess the effect of MTGC on early LV function changes in such a large cohort of very well-phenotyped healthy volunteers and non-healthy subjects without heart failure.

The main findings of this study were: 1) among the many clinical and biological parameters studied, BMI was the only independent correlate of MTGC; 2) MTGC was independently associated with LV volumes and LV mass; 3) MTGC was not independently associated with systolic and diastolic strain measures.

As previously reported (12, 33), MTGC was positively associated with age, BMI, and waist circumference. As expected, subjects in the highest MTGC quartile were more likely to be living with T2D than subjects in the lowest MTGC quartile, which confirms previous data showing that cardiac ectopic fat and in particular myocardial fat increases with diabetes. This is in line with previous studies demonstrating that T2D patients have significantly higher MTGC than healthy individuals, independent of age or BMI (14, 34, 35).

In multivariate regression analyses, only BMI was found to be an independent determinant of MTGC, but all the parameters included in the analysis together explained only 20% of the MTGC variance, suggesting that obesity per se is not sufficient to cause myocardial steatosis. In addition, 14 healthy volunteers were found to belong to the high MTGC quartile subgroup (MTGC > 75^th^ percentile), which highlights the hypothesis that BMI alone cannot predict MTGC and that it is therefore important to look further than BMI alone to phenotype ectopic fat depots in patients living with metabolic disease and unhealthy obesity.

Studies conducted in healthy volunteers have shown that a short-term very-low-calorie diet leads to an accumulation of myocardial triglyceride (36, 37) and that progressive caloric restriction induces a dose-dependent increase in MTGC (38). Conversely, in patients living with T2D and obesity, prolonged caloric restriction may be associated with a reduction of MTGC (39). These observations are consistent with the hypothesis that MTGC could act as the fuel of myocardium in healthy subjects under stress conditions, in particular in young athletes, and could be modulated by lifestyle changes and be more lipotoxic in metabolic disease subjects. MTGC could thus be considered as a highly flexible source of free fatty acids *via* lipolysis to the myocardium, but chronic accumulation of triglycerides in the myocardium could drive an overload of toxic lipid intermediates that could, along with other processes such as inflammation or fibrosis, contribute to LV dysfunction (40, 41). We and others have already shown that like myocardial steatosis, this flexibility i.e. ability to mobilize ectopic fat depots are less manifest in patients with severe obesity who lose weight following bariatric surgery (23, 42). On the other hand, the risk of MTGC accumulation should also be explained by other factors, and notably a genetic component. Genetic polymorphisms may influence susceptibility to develop a chronic imbalance of lipid storage versus lipid oxidation. Therefore, genetic studies are necessary to gain deeper insight into the pathogenesis of this myocardial steatosis, as had already been done for other diseases involving ectopic fat deposition, such as metabolic-associated fatty liver disease (43–45).

Multiple animal studies (9, 10) support the concept that myocardial steatosis can lead to LV dysfunction. They posit that myocardial accumulation of toxic fatty acid intermediates entails cellular damage, apoptosis and replacement fibrosis, leading to contractile LV dysfunction (9, 46, 47). Some ^1^H-MRS human studies report similar associations (48–51), but our results give a more nuanced picture. We found that LV volumes were negatively associated with MTGC. This is in agreement with data that we and others had previously published (12, 15). Furthermore, we found that higher LV mass was correlated with higher MTGC. Szczepaniak et al. (52) found a similar association in 15 healthy lean subjects, which is in line with animal studies that suggest ceramide accumulation in nonadipocyte cells could promote hypertrophic signaling (9). However, the association between LV mass and MTGC was less strong in obesity. Indeed, in multivariate analysis, we found that LV mass was independently associated with MTGC, but in obese subjects, if there was high MTGC, then LV mass was not as high as in lean subjects.

For several years now, myocardial strain and strain rates have emerged as key parameters for detecting subclinical alteration in LV function, and are now displacing conventional cardiac function parameters for LV function analysis (53–55). Myocardial strain assesses myocardial deformation and can easily be measured by routine echocardiography and more recently by MRI. Studies show that it is more sensitive than traditional imaging markers for detecting early myocardial injury and predicting major cardiac events (56–58). After acute myocardial infarction, it also has better prognostic value than conventional LV function parameters (59). Strain imaging is now widely used in clinical practice and in clinical research, and is beginning to find use in MTGC studies.

Regarding systolic strain, very few studies have found an association between MTGC and LV systolic dysfunction, and only a handful of them performed a strain measurement (50, 51, 60). Mahmod et al. found that MTGC was an independent determinant of impaired circumferential and longitudinal systolic LV strain assessed by cardiac MRI tagging (60). In a more recent cardiac MRI study on 53 patients living with T2D and 20 healthy subjects, Gao et al. (51) showed that increased MTGC was independently associated with impaired longitudinal systolic strain. Contrary to these studies, we did not find an association between increased MTGC and impaired systolic strain. It is possible that because we included only patients without overt criteria of heart failure, we could not observe an association between MTGC and early LV systolic dysfunction. Longitudinal studies including different stages of heart failure (preserved or reduced) and different time intervals for assessment of LV function parameters would help to detect changes in LV function and establish whether and how these changes are related to MTGC.

MTGC was correlated to diastolic function abnormalities, and notably with the global peak early diastolic circumferential strain rate, which is one of the main parameters currently used to study diastolic function in MTGC studies (49, 61). In multivariate analysis, MTGC remained independently associated with end-diastolic volume but not with other parameters of diastolic dysfunction, except for global peak late diastolic longitudinal strain rate (LDSRl) that remained positively associated to MTGC. However, MTGC explained only 18% of the LDSRl variance, and we cannot reach firm conclusions based solely on this one parameter because it probably reflects a compensatory mechanism that can be related to the late atrial ventricular filling velocity (peak A-wave velocity). Our results contrast with previous studies that have shown a linear association between MTGC and impaired LV diastolic function (14, 16, 62, 63). Rijzewijk et al. (14) demonstrated that increased MTGC was associated with impaired mitral E/A ratio and impaired peak E-wave velocity. However, the sensitivity of this technique for detecting diastolic dysfunction is not perfect, notably because there is evidence of a pseudonormal E/A pattern related to diastolic dysfunction (64, 65).

Regarding early diastolic dysfunction, myocardial steatosis was also found to be independently associated with impaired diastolic strain rate. Ng et al. (62) studied 42 men living with T2D and found an independent association between MTGC and early diastolic strain rate measured by echocardiography. Similarly, Korosoglou et al. (16) found an independent association between MTGC and early diastolic strain rate measured by MRI.

Nyman et al. found that MTGC was not a determinant of diastolic function, whereas it was correlated with early LV diastolic dysfunction in univariate analysis (15). Likewise, in a cohort of 75 nondiabetic men, Graner et al. failed to find any association between MTGC and diastolic function, but note that they did not use strain to assess LV function (66).

Patients living with T2D or obesity have traditionally been characterized as having diastolic dysfunction with normal systolic function (35, 67). While some authors have suggested that MTGC is involved in this pathological process (14, 16, 33), our results and others offer a more nuanced picture. In a MRI myocardial tagging study, MTGC was independently associated to global peak systolic longitudinal strain but was not correlated at all to diastolic strain rate (50), which illustrates, once again, that the association between MTGC and diastolic dysfunction is not unequivocal. As we demonstrated earlier, data on the effect of MTGC on LV function remains inconsistent. We can speculate that associations between MTGC and impaired LV function are likely to be indirect, and that other unmeasured pathologic processes may be more directly responsible for LV dysfunction in patients living with T2D or obesity. Furthermore, only LV volumes (EDV and ESV) remained independently associated with MTGC in our multivariate analysis. We can hypothesize that, contrary to previous work, MTGC does not affect the fitting of early LV function abnormalities, but might worsen a pre-existing phenomenon. Indeed, there are other mechanisms that appear to be intricately linked with cardiac lipotoxicity and that may also promote cardiac cellular damage, such as glucotoxicity which is also described as being associated with myocardial dysfunction (68–70). Thus, although metabolic channeling of excess fatty acids to intracellular triacylglycerol stores may serve an initial cytoprotective role by sequestering excess fatty acids away from mitochondria, endoplasmic reticulum and other organelles, the capacity for physiological storage of lipids in the heart is limited (71). The endogenous mechanisms that determine the transition between adaptive lipid storage and lipotoxicity are still not well understood.

This work has some strengths. It was performed in a large cohort of subjects, whereas to our knowledge, most previous MTGC studies were performed on small cohorts with less than a hundred subjects. Moreover, we only included subjects who were free of heart failure, in order to analyze the early abnormalities of LV function. Regarding strain measurement, we used MRI-FT, which is a promising tool with good feasibility and reproducibility for global strain measurement (72). In addition, the strain values found here were in line with values reported in some prior MRI-FT studies (29, 73–75). Finally, healthy subjects were rigorously phenotyped, including for any cardiovascular disease or comorbidities that could impact LV function (notably diabetes, obesity, dyslipidemia, or hypertension).

However, this study has also some limitations. First, other biomarkers could have been considered to predict MTGC, such as β-hydroxybutyrate, a ketone body that may be inversely associated with increased MTGC (76). Unfortunately, the retrospective design of our study made it impossible to include additional biological parameters that had not been measured in each cohort’s original study design. Second, we used non-invasive MRS to quantify the intramyocardial triglyceride content without performing confirmatory histological examination. Nevertheless, ^1^H-MRS is an established non-invasive method that has demonstrated high diagnostic accuracy for the assessment of myocardial steatosis (77). Third, as we conducted an observational cross-sectional study, we were unable to observe a deterioration in LV function, which may have limited our capacity to observe a potential relationship between MTGC and LV function. Fourth, among the subjects with high MTGC (MTGC >75th percentile), 61.9% had T2D, suggesting that this population had both glucotoxicity and lipotoxicity.

## Conclusion

In conclusion, in a large cohort of healthy and non-healthy subjects, only BMI remained independently associated with increased MTGC in multivariate analysis, which suggests the existence of unknown factors to predict the rise of MTGC. Further prospective studies are required in order to identify these factors.

Using MRI to study myocardial triglyceride content and systolic and diastolic LV function simultaneously with MRI-FT tracking for strain measurement, we found that MTGC is associated with LV dysfunction. However, this relationship is non-linear, and MTGC does not appear to be involved in early changes in LV function. The impact of myocardial steatosis on LV function therefore remains a matter of ongoing debate, and future longitudinal studies are needed in a large cohort of healthy and non-healthy age-matched subjects in order to conclusively determine whether increased MTGC is associated with LV dysfunction.

## Data availability statement

The original contributions presented in the study are included in the article/**Supplementary Material**. Further inquiries can be directed to the corresponding author.

## Ethics statement

The studies involving human participants were reviewed and approved by NCT01284816, NCT02042664, NCT03118336. The patients/participants provided their written informed consent to participate in this study.

## Author contributions

All authors contributed to the article and approved the submitted version.
