# Supplementary material for: Is increased myocardial triglyceride content associated with early changes in left ventricular function? A 1H-MRS and MRI strain study
Source: Front Endocrinol (Lausanne). 2023 Jun 22;14:1181452. doi: 10.3389/fendo.2023.1181452 (PMC10323751; doi:10.3389/fendo.2023.1181452)
Supplement: Supplementary file 1 [file Table_1.docx]

**SUPPLEMENTARY**

**Table 1: Comparison of conventional left ventricular parameters between healthy subjects and patients living with type 2 diabetes or obesity (n=336)**

|  | **Healthy subjects (n=206)** | **Patients living with T2D and/or obesity  (n=130)** | **p value** |
| --- | --- | --- | --- |
| **MTGC (%)** | 0.42 [0.23;0.67] | 1.2 [0.6;1.82] | *< 0.0001* |
| ***Cardiac geometry*** | | | |
| **LV mass (g)** | 87.6 [69.3;111.5] | 114.7 [96.1;142.4] | *< 0.0001* |
| **LV mass index (g/cm²)** | 50.9 [43.8;60.3] | 56.8 [48.3;63.9] | *< 0.0001* |
| ***Conventional LV function parameters*** | | | |
| **LV mass/EDV (g/mL)** | 0.64 [0.59;0.71] | 0.92 [0.79;1.07] | *< 0.0001* |
| **Cardiac output (L/min)** | 6.02 [5.3;7.1] | 6.3 [5.3;7.3] | 0.22 |
| **Cardiac index (L/min/m²)** | 3.5 [3.1;3.9] | 2.9 [2.6;3.5] | *< 0.0001* |
| ***Conventional LV systolic function parameters*** | | | |
| **LVEF (%)** | 65.9 [60.9;69.7] | 68.2 [60.5;73.3] | *0.018* |
| **ESV (mL)** | 46.7 [35.6;57.9] | 39.9 [31;54.5] | *0.001* |
| **ESV index (mL/m²)** | 26.7 [22.2;31.4] | 18.7 [15.6;25.3] | *< 0.0001* |
| **SV (mL)** | 89 [74.2;108.1] | 82.9 [70.9;96.2] | *0.011* |
| **SV index (mL/m²)** | 51.6 [47.8;58] | 40.7 [35.5;46.4] | *< 0.0001* |
| ***Conventional LV diastolic function parameters*** | | | |
| **EDV (mL)** | 138.3 [112.8;164] | 122.9 [107.6;151.8] | *0.027* |
| **EDV index (mL/m²)** | 79.3 [69.7;86.7] | 60.9 [53.3;68.6] | *< 0.0001* |
| **Peak E velocity (cm/s)** | 73.5 [64.4;82.6] | 52.3 [43.2;64.6] | *< 0.0001* |
| **Peak A velocity (cm/s)** | 32.3 [26.9;37.9] | 42.3 [33.7;52.5] | *< 0.0001* |
| **Mitral E/A ratio** | 2.3 [1.8;2.8] | 1.3 [1.03;1.6] | *< 0.0001* |

*Data expressed as mean ± standard deviation (SD) or as median [25^th^ percentile;75^th^ percentile]
MTGC: myocardial triglyceride content; LV: left ventricular; LVEF: left ventricular ejection fraction; SV: stroke volume; ESV: end-systolic volume; EDV: end-diastolic volume; T2D: type 2 diabetes*

**Table 2: Comparison of strain parameters between healthy subjects and patients living with type 2 diabetes or obesity (n=323)**

|  | **Healthy subjects (n=196)** | **Patients living with T2D and/or obesity  (n=127)** | **p value** |
| --- | --- | --- | --- |
| **GCS (%)** | -20.3 [-22;-18.6) | -20.29 [-22.03;-17.96] | 0.84 |
| **GLS (%)** | -17.8 [-19.8;-15.9] | -16.8 [-19.7;-14.8] | 0.07 |
| **SRc (s^-1^)** | -1.08 [-1.2;-0.97] | -1.1 [-1.3;-0.98] | *0.08* |
| **SRl (s^-1^)** | -0.86 [-0.99;-0.74] | -0.90 [-1.01;-0.76] | 0.30 |
| **EDSRc (s^-1^)** | 1.05 (0.91;1.20) | 0.82 (0.69;0.99) | *< 0.0001* |
| **LDSRc (s^-1^)** | 0.22 (0.17;0.28) | 0.47 (0.31;0.69) | *< 0.0001* |
| **EDSRl (s^-1^)** | 0.81 (±0.24) | 0.76 (±0.22) | 0.08 |
| **LDSRl (s^-1^)** | 0.37 (0.29;0.48) | 0.47 (0.36;0.56) | *< 0.0001* |

*Data expressed as mean ± standard deviation (SD) or as median [25^th^ percentile;75^th^ percentile]

GCS: global peak systolic circumferential strain; GLS: global peak systolic longitudinal strain; SRc: global peak systolic circumferential strain rate; SRl: global peak systolic longitudinal strain rate; EDSRc: global peak early-diastolic circumferential strain rate; EDSRl: global peak early-diastolic longitudinal strain rate; LDSRc: global peak late-diastolic circumferential strain rate; LDSRl: global peak late-diastolic longitudinal strain rate; T2D: type 2 diabetes*

**Table 3: Comparison of strain parameters between subjects with low *versus* high myocardial triglyceride content**

|  | **Low MTGC ≤ 0.31%**  **n = 81** | **High MTGC >1.09%**  **n = 82** | **p value** |
| --- | --- | --- | --- |
| **GCS (%)** | -20.4 (±2.9) | -20.7 (±3.1) | 0.447 |
| **GLS (%)** | -17.7 (±2.7) | -17.6 (±2.7) | 0.852 |
| **SRc (s^-1^)** | -1.1 [-1.2;-1.0] | -1.1 [-1.3;-1.0] | 0.058 |
| **SRl (s^-1^)** | -0.8 [-1.0;-0.7] | -0.9 [-1.0;-0.8] | *0.019* |
| **EDSRc (s^-1^)** | 1 (±0.2) | 0.9 (±0.2) | *0.001* |
| **LDSRc (s^-1^)** | 0.2 [0.2;0.3] | 0.5 [0.3;0.7] | *<0.0001* |
| **EDSRl (s^-1^)** | 0.8 (±0.2) | 0.7 (±0.2) | 0.116 |
| **LDSRl (s^-1^)** | 0.4 [0.3;0.5] | 0.5 [0.4;0.6] | *<0.0001* |

*Data expressed as mean ± standard deviation (SD) or as median [25^th^ percentile;75^th^ percentile]*

*MTGC: myocardial triglyceride content; GCS: global peak systolic circumferential strain; GLS: global peak systolic longitudinal strain; SRc: global peak systolic circumferential strain rate; SRl: global peak systolic longitudinal strain rate; EDSRc: global peak early-diastolic circumferential strain rate; EDSRl: global peak early-diastolic longitudinal strain rate; LDSRc: global peak late-diastolic circumferential strain rate; LDSRl: global peak late-diastolic longitudinal strain rate*

**Table 4: Comparison of clinical and biological data between subjects with low versus high myocardial triglyceride content in a subgroup analysis (healthy subjects compared to patients living with T2D and/or obesity patients)**

|  | **Healthy subjects** | | | **Patients living with T2D and/or obesity** | | |
| --- | --- | --- | --- | --- | --- | --- |
|  | **Low MTGC ≤ 0.31% n=52** | **High MTGC  > 0.90% n=52** | **p value** | **Low MTGC ≤ 0.59% n=32** | **High MTGC > 1.83% n=32** | **p value** |
| ***Clinical data*** | | | | | | |
| **Sex ratio: women (n. %)** | 27 (51.9%) | 29 (55.8%) | 0.78 | 19 (59.4%) | 21 (65.6%) | 0.72 |
| **Age (years)** | 22.0 [21.0;25.0] | 21.0 [20.0;24.0] | 0.4 | 50.5 [38.9;60.0] | 53.0 [42.0;61.2] | 0.62 |
| **BMI (kg/m²)** | 21.0 [19.3;23.1] | 21.5 [20.2;23.8] | 0.21 | 38.1 [31.8;44.6] | 35.8 [31.8;41.6] | 0.43 |
| **T2D (n. %)** | 0 (0.0%) | 0 (0.0%) | 1 | 24 (75.0%) | 26 (81.2%) | *0.023* |
| **WC (cm)** |  |  |  |  |  |  |
| **-women** | 69.5 [64.2;74.0] | 70.0 [67.0;75.0] | 0.57 | 122.0 [114.5;130] | 114.0 [102.0;123] | 0.065 |
| **-men** | 77.0 [74.0;83.0] | 80.5 [76.2;84.8] | 0.28 | 111.0 [105;144] | 111.0 [99.5;125.0] | 0.31 |
| **Obesity (n. %)** | 0 (0 %) | 0 (0.0%) | 1 | 22 (68.8%) | 18 (56.2%) | 0.27 |
| **Dyslipidemia  (n. %)** | 0 (0 %) | 0 (0 %) | 1 | 18 (56.2%) | 20 (62.5%) | 0.47 |
| **Arterial hypertension  (n. %)** | 0 (0 %) | 0 (0 %) | 1 | 18 (56.2%) | 18 (56.2%) | 1 |
| ***Biological data*** | | | | | | |
| **Total cholesterol (mmol/L)** | 1.7 [1.5;1.8] | 1.8 [1.6;2] | *0.023* | 1.6 [1.4;1.8] | 1.7 [1.4;2.0] | 0.25 |
| **Triglycerides (mmol/L)** | 0.6 [0.5;0.9] | 0.8 [0.6;0.9] | 0.12 | 1.4 [1.0;2.1] | 1.3 [1.0;1.6] | 0.6 |
| **HDL (mmol/L)** | 0.5 [0.5;0.7] | 0.6 [0.5;0.6] | 0.88 | 0.4 [0.3;0.5] | 0.4 [0.4;0.5] | 0.58 |
| **LDL (mmol/L)** | 1.0 (±0.3) | 1.1 (±0.3) | 0.079 | 0.9 (±0.3) | 1.0 (±0.3) | 0.42 |
| **Fasting plasma glucose (mmol/L)** | 4.7 [4.4;5.0] | 4.7 [4.5;4.9] | 0.42 | 6.7 [5.8;10.2] | 7.7 [6.2;10.0] | 0.46 |
| **Fasting plasma insulinemia (mUI/L)** | 6.7 [5.0;8.9] | 7.2 [5.3;8.9] | 0.35 | 21.6 [15.8;23.9] | 14.2 [12.4;19.2] | 0.092 |
| **HOMA-IR** | 1.4 [1.0;1.9] | 1.5 [1;1.8] | 0.68 | 5.5 [3.4;7.8] | 4.5 [3.0;6.6] | 0.42 |

*Low MTGC was defined as MTGC ≤25th percentile, and high MTGC as MTGC >75th percentile of each subgroup. Data expressed as mean ± standard deviation (SD) or as median [25^th^ percentile;75^th^ percentile]*
*BMI: body mass index; HOMA-IR: homeostatic model assessment of insulin resistance; HDL: high-density lipoprotein; LDL: low-density lipoprotein; T2D: type 2 diabetes; WC: waist circumference*

**Table 5: Comparison of conventional left ventricular parameters between subjects with low versus high myocardial triglyceride content in a subgroup analysis (healthy subjects compared to patients living with T2D and/or obesity patients)**

|  | **Healthy subjects** | | | **Patients living with T2D and/or obesity** | | |
| --- | --- | --- | --- | --- | --- | --- |
|  | **Low MTGC  ≤ 0.31% n=52** | **High MTGC  > 0.90% n=52** | **p value** | **Low MTGC**  **≤ 0.59% n=32** | **High MTGC**  **> 1.83% n=32** | **p value** |
| ***Cardiac geometry*** | | | | | | |
| **LV mass (g)** | 85.5 [72.1;108.8] | 84.3 [70.7;107.5] | 0.75 | 127.9 [103.0;148.5] | 104.2 [95.9;124.1] | *0.024* |
| **LV mass index (g/cm^2^)** | 52.2 (±10.3) | 51.2 (±12.3) | 0.64 | 61.4 (±13.9) | 55.4 (±13.7) | 0.087 |
| ***Conventional LV function parameters*** | | | | | | |
| **LV Mass/EDV (g/mL)** | 0.6 [0.6;0.7] | 0.6 [0.6;0.7] | 0.55 | 0.9 [0.8;1.1] | 0.9 [0.8;1.0] | 0.76 |
| **Cardiac output (L/min)** | 6.0 (±1.5) | 6.2 (±1.4) | 0.47 | 6.9 (±1.8) | 6.2 (±1.6) | 0.13 |
| **Cardiac index (L/min/m²)** | 3.4 (±0.7) | 3.5 (±0.6) | 0.51 | 3.2 (±0.7) | 3.1 (±0.7) | 0.53 |
| ***Conventional LV systolic function parameters*** | | | | | | |
| **LVEF (%)** | 65.5 (±6.8) | 65.5 (±6.0) | 1 | 65.6 (±9.0) | 69.6 (±8.6) | 0.072 |
| **ESV (mL)** | 43.8 [34.9;58.5] | 46.9 [35.3;55.4] | 0.96 | 44.4 [33.9;61.6] | 35.9 [28.4;43.7] | *0.025* |
| **ESV index (mL/m²)** | 25.3 [21.7;32.6] | 26.4 [23.3;30.2] | 0.86 | 21.0 [16.7;27.5] | 17.9 [15.2;22.1] | 0.08 |
| **SV (mL)** | 91.7 (±23.2) | 89.6 (±20.2) | 0.63 | 89.1 (±21.2) | 84.5 (±20.2) | 0.37 |
| **SV index (mL/m²)** | 52.3 (±9.4) | 50.8 (±8.7) | 0.38 | 41.5 (±8.0) | 41.9 (±8.2) | 0.87 |
| ***Conventional LV diastolic function parameters*** | | | | | | |
| **EDV (mL)** | 128.7 [112.9;171.5] | 143.9 [113.5;158.6] | 0.8 | 140.5 [112.6;161.5] | 116.1 [107.9;137.3] | *0.045* |
| **EDV index (mL/m²)** | 80.0 (±15.4) | 77.7 (±10.8) | 0.38 | 64.1 (±13.1) | 61.2 (±15.2) | 0.42 |
| **Peak E velocity (cm/s)** | 72.1 (±11.7) | 74.9 (±12.2) | 0.25 | 52.8 (±21.0) | 60.3 (±16.5) | 0.16 |
| **Peak A velocity (cm/s)** | 31.2 (±8.8) | 34.6 (±10.3) | 0.084 | 42.0 (±11.8) | 44.4 (±15.3) | 0.52 |
| **Mitral E/A ratio** | 2.4 [1.8;2.8] | 2.3 [2.0;2.8] | 0.47 | 1.3 [1.0;1.6] | 1.3 [1.1;1.7] | 0.27 |

*Low MTGC was defined as MTGC ≤25th percentile, and high MTGC as MTGC >75th percentile of each subgroup. Data expressed as mean ± standard deviation (SD) or as median [25^th^ percentile;75^th^ percentile]

MTGC: myocardial triglyceride content; LV: left ventricular; LVEF: left ventricular ejection fraction; SV: stroke volume; ESV: end-systolic volume; EDV: end-diastolic volume; T2D: type 2 diabetes*
